# Electronic structure and low-temperature thermoelectric transport of TiCoSb single crystals†

**DOI:** 10.1039/d2nr02556f

**Published:** 2022-06-21

**Authors:** Federico Serrano-Sanchez, Mengyu Yao, Bin He, Dong Chen, Andrei Gloskovskii, Alexander Fedorov, Gudrun Auffermann, Enke Liu, Ulrich Burkhardt, Gerhard H. Fecher, Chenguang Fu, Claudia Felser, Yu Pan

**Affiliations:** Max Planck Institute for Chemical Physics of Solids 01187 Dresden Germany Serrano@cpfs.mpg.de Yu.Pan@cpfs.mpg.de; Deutsches Elektronen-Synchrotron DESY 22607 Hamburg Germany; Helmholtz-Zentrum Berlin für Materialien und Energie Berlin Germany; Institute for Solid State Research, Leibniz IFW Dresden 01069 Dresden Germany; Beijing National Laboratory for Condensed Matter Physics, Institute of Physics, Chinese Academy of Sciences Beijing 100190 P. R. China; State Key Laboratory of Silicon Materials, School of Materials Science and Engineering, Zhejiang University 310027 Hangzhou China

## Abstract

Band structure engineering has a strong beneficial impact on thermoelectric performance, where theoretical methods dominate the investigation of electronic structures. Here, we use angle-resolved photoemission spectroscopy (ARPES) to analyze the electronic structure and report on the thermoelectric transport properties of half-Heusler TiCoSb high-quality single crystals. High degeneracy of the valence bands at the L and Γ band maximum points was observed, which provides a band-convergence scenario for the thermoelectric performance of TiCoSb. Previous efforts have shown how crystallographic defects play an important role in TiCoSb transport properties, while the intrinsic properties remain elusive. Using hard X-ray photoelectron spectroscopy (HAXPES), we discard the presence of interstitial defects that could induce in-gap states near the valence band in our crystals. Contrary to polycrystalline reports, intrinsic TiCoSb exhibits p-type transport, albeit defects still affect the carrier concentration. In two initially identical p-type TiCoSb crystal batches, distinct metallic and semiconductive behaviors were found owing to defects not noticeable by elemental analysis. A varying Seebeck effective mass is consistent with the change at the Fermi level within this band convergence picture. This report tackles the direct investigation of the electronic structure of TiCoSb and reveals new insights and the strong impact of point defects on the optimization of thermoelectric properties.

## Abstract 2

The electronic structure of half-Heusler TiCoSb single crystals reveals a band-convergence scenario in which slight changes at the Fermi level lead to significant differences in the Seebeck effective mass and electronic properties.

## Graphical abstract

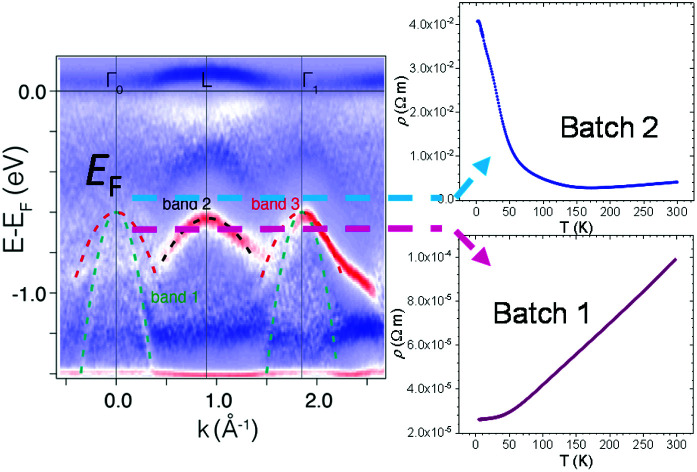

## Introduction

Thermoelectric materials display the exceptional ability to convert thermal gradients into electrical energy. In the last decade, emerging approaches have brought forth enhanced thermoelectric performance owing to the investigations on the intrinsic electronic structure of materials.^1–4^ Among those, band engineering, which involves tuning of the electronic band structure by resonant levels or band convergence, targets at improved power factor, *S*^2^*σ*, where *S* is the Seebeck coefficient and *σ* is the electrical conductivity, increasing the efficiency of thermoelectric materials. Specific electronic structural parameters have gained relevance, such as the band effective mass (m*), valley degeneracy (*N*_v_), band anisotropy, and band-gap energy (*E*_g_), for their role in the thermoelectric transport properties. By manipulating these parameters, new possibilities for the further development of highly efficient materials have emerged. Thus, direct observation and investigation of these materials using spectroscopic techniques, such as angular-resolved photoemission spectroscopy (ARPES), is crucial for supporting advancements in this field.

Half-Heusler alloys are among the most promising thermoelectric materials that exhibit high performance at high temperatures. With a variety of compositions and complex band structures, they exhibit a prominent range of physical properties such as excellent optical and magnetic properties, heavy fermions, and superconductivity.^5^ These alloys are typically understood through the valence electron count (VEC) of their composition elements that have the general formula *XYZ*, in which *X* and *Y* are electropositive elements, which are commonly transition metals, and *Z* is an electronegative element, often a group 14 or 15 element. The high thermoelectric performance, robust mechanical and thermal stability, and non-toxic compositions of semiconductive half-Heusler alloys with 8 or 18 valence electrons are the attractive properties that give prominence to promising thermoelectric candidates.^6–15^ Extensive investigations reveal that the disorder and defects in the TiCoSb *C*1_b_ type structure are still under debate.^16–18^ Vacant site occupational defects and crystallographic disorder are recognized as the main factors determining the electrical transport in this material, yielding in-gap states, varying the Fermi level (*E*_F_), altering the band structure, and acting as scattering centres. Thus, the reported properties of TiCoSb showed significant differences depending on its preparation conditions.^17,19–24^ Moreover, the defective nature of this system has led to discrepancies in the theoretical calculations of the band-gap energy and electronic structure, showing a different *E*_g_ value compared to the experimental value.^25,26^ These issues can be better clarified by investigating single crystals, which display the intrinsic features that contribute to thermoelectric performance.^27^ Therefore, the direct experimental observation of the intrinsic transport and band structure of TiCoSb single crystals using ARPES was essential to provide a direct measurement of the electronic band structure of TiCoSb and to understand the mechanisms behind its high thermoelectric performance.

In the present study, we investigated the electronic structure and point defect chemistry of TiCoSb single crystals by ARPES and hard X-ray photoelectron spectroscopy (HAXPES) techniques along with their thermoelectric transport properties. High-quality homogeneous TiCoSb single crystals were grown using the flux method. ARPES displayed a blurred surface state above the valence band maximum and a rather small energy difference between the L and Γ band maxima, which yields a band convergence picture. No in-gap states were detected in the bulk electronic structure under either ARPES or HAXPES. Nevertheless, two different transport behaviors were found for different crystal batches prepared under the same conditions. A small defect difference significantly alters the behavior of the samples from metallic to semiconducting. The Hall effect and Seebeck coefficient measurements indicated that TiCoSb single crystals have intrinsic p-type transport, contrary to the n-type character of undoped polycrystalline samples reported to date. Consequently, we determined that the thermoelectric performance of TiCoSb is drastically changed by small differences in its crystallographic properties, and high-band degeneracy promotes high efficiency in p-type doped derivatives.

## Experimental

### Single-crystal growth of TiCoSb

Single crystals of TiCoSb were grown using the Sb flux method. High-purity Ti (Alfa Aesar, 99.99% metal basis) and Co (Alfa Aesar, 99.998% metal basis) powders were mixed with Sb shots (Alfa Aesar, 99.999% metal basis) in a 3 : 3 : 10 molar ratio and loaded into an Al_2_O_3_ crucible. The crucible was covered with a small piece of glass wool filter and sealed in a quartz tube under high vacuum. Then, the ampoule was placed into a furnace and heated up to 1150 °C at 100 °C h^−1^, dwelled for 24 h, and slowly cooled down to 900 °C at 1.5 °C h^−1^ with a final dwell of another 24 h. Subsequently, the ampoule was cooled down to 760 °C and the excess Sb was removed by centrifugation. Sb impurities and negligible amounts of CoSb were found on the surface of the as-grown crystals, which were removed by polishing or diluted by HCl : HNO_3_ acidic treatment.

### Crystallinity and composition analyses of TiCoSb single crystals

The crystallinity and orientation of the samples were determined using a Laue X-ray diffractometer (XRD). Several pieces of the crystals were hand-ground in an agate mortar and analysed by XRD using a Huber G670 imaging-plate Guinier powder diffraction camera—which was exposed to Cu-K_α1_ radiation—equipped with a Ge(111) monochromator. Rietveld refinements of the XRD patterns were performed using FullProf software.^28,29^ A pseudo-Voigt function was employed to analyse the shapes of the diffraction peaks; there were no regions excluded from the refinements.

The microscopic morphology of the crystals was observed using the backscattered electron mode under a scanning electron microscope (SEM; Zeiss Merlin SEM, Carl Zeiss AG, Oberkochen, Germany) at an accelerating voltage of 16 kV and a beam current of 2 nA. Crystal elemental quantitative electron probe microanalysis was performed using an EDX spectroscopy analyser (Phoenix V 5.29, EDAX) and a WDX electron microprobe (Cameca SX100) using Ti and CoSb as standards.

### HAXPES experimental setup

HAXPES measurements were conducted at beamline P22 which was built at PETRA III (Hamburg, Germany).^30,31^ The photon energy was set to *hν* = 6000 eV using a Si(111) high-heat-load double-crystal primary monochromator and the (333) reflection of a Si double channel-cut four-bounce post monochromator. The degree of polarization was approximately 98%.^32^ The photon incidence nearly grazed at *α* = 89°. The Fermi energy *ε*_F_ was obtained at a kinetic energy of *E*_kin_ = 6000.50 eV with a width of 200 meV. Accounting for the 28 meV thermal broadening of the Fermi–Dirac distribution at room temperature (300 K), this corresponded to an overall energy resolution of approximately 170 meV (*E*/Δ*E* ≈ 3.5 × 10^4^).

### ARPES experimental setup

Experiments were performed at the Berliner Elektronenspeicherring für Synchrotronstrahlung (BESSY) (beamline UE112-PGM-1) using a Scienta Omicron R8000 analyser. Single-crystal samples of batch 1 were cleaved *in situ* at 15 K. The base pressure was less than 1 × 10^−10^ mbar. ARPES spectra were acquired with a linear horizontal polarization photon of *hν* = 130 eV. The system was configured with an angular mode of 30 and a pass energy of 20, which led to an overall energy resolution of ∼15 meV and a spatial resolution greater than 0.1°. ARPES experiments were performed on a bar-shaped sample oriented along the [110] direction. A small perpendicular incision was made with a 50 μm wire saw to facilitate cleavage, yielding a shiny and high-quality surface for spectroscopic measurement, as previously demonstrated in the ARPES study of ZrNiSn.^27^

### Transport measurements

The longitudinal resistance and Hall coefficient were measured using a four-probe method with the electrical transport option of a physical property measurement system (PPMS). The Hall carrier concentration was determined using the formula *n*_H_ = *1*/(*eR*_H_), where *R*_H_ is the Hall coefficient and *e* is the elementary unit charge. The Seebeck coefficient (*S*) and thermal conductivity (*κ*) were measured using a one-heater two-thermometer set-up under high vacuum in a PPMS with type-E thermocouples as the thermometers. The thermoelectric voltages were measured using a Keithley 2182A nanovoltmeter. The field dependence of the thermocouples can be negligible within the temperature range of interest.

## Results and discussion

### TiCoSb single-crystal characterization and ARPES electronic structure

**Fig. 1:**
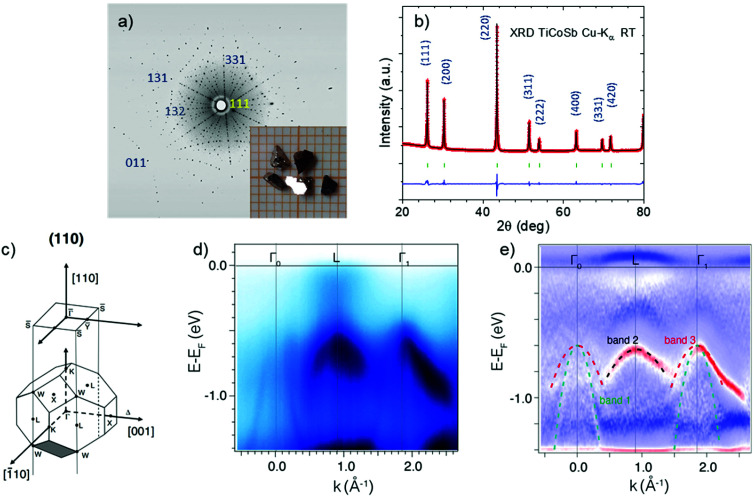
(a) Laue diffraction pattern along the [111] direction. The inset shows a digital picture of the TiCoSb crystals. (b) XRD Rietveld refinement of the TiCoSb crystal powder pattern. (c) Bulk Brillouin zone including high-symmetry points with the projected (110) *k*_*y*_ − *k*_*x*_ surface highlighted. (d) ARPES measurement of the band dispersion along the Γ–L–Γ symmetry points and (e) derivative of data in (c) with a clearer display of the valence bands. Lines above the *E*_F_ are artefacts of data processing.

In previous studies based on polycrystalline samples, the occupation by Ti or Co of the vacant tetrahedral 4d site in the *F*4̄3*m* crystalline structure generated in-gap states in TiCoSb, as confirmed by HAXPES measurements of arc-melted TiCoSb.^26^ The nature of these states depends on the interstitial element; in the case of Ti interstitials, new localized bands are near the conduction band minimum, whereas for Co interstitials, the impurity band is located near the valence band maximum. These varied point defects strongly alter the carrier concentration, resulting in significant sample-to-sample variations in the thermoelectric properties of the material.^15,17,20,26,33^ To investigate how the electronic band structure is affected by point defect chemistry, and therefore, shed light on strategies to further improve the thermoelectric performance of TiCoSb systems, high-quality TiCoSb single crystals were grown for ARPES and HAXPES experiments and for measurement of the transport properties. The as-grown TiCoSb single crystals had a length of 1–3 mm and a shiny surface, showing the typical triangular pyramidal shape of half-Heusler alloys with preferential growth along the (111) surface. Fig. 1a shows the Laue diffraction pattern along the [111] direction with the inset of an optical picture of the TiCoSb crystals. The Laue diffraction pattern indexed in the cubic *F*4̄3*m* space group matched the calculated profile of the crystal along the [111] direction and confirmed crystallinity across the samples.

The XRD patterns of the crystal powders (Fig. 1b) were used to assess the purity of the samples and confirm the absence of other impurities, which was also evidenced by the homogeneous polished faces of the crystals in the SEM images (Fig. S1†). The Rietveld refinement (Table S1†) of the pattern displayed a slightly off-stoichiometry; nevertheless, wavelength-dispersive spectroscopy (Table S2†) demonstrated an almost stoichiometric 1 : 1 : 1 composition. Chemical composition analyses were performed on crystals from different batches, which were prepared under identical conditions, to confirm the reproducibility of the synthesis and compare the properties of the crystals. Compositional analyses revealed that the crystals were highly stoichiometric. Thus, the off-stoichiometry of the occupancy factors in the XRD refinement may have resulted from anti-site defects or occupation of the vacant 4d position defects in the crystal, which did not alter the overall composition of the samples but depended on the crystallographic sites.

In addition to the most commonly adopted theoretical calculation methods, the ARPES technique can experimentally determine the band structure, but requires high-quality single crystals. Herein, TiCoSb single crystals were analyzed by ARPES to directly observe their electronic band structure, and the results were compared with DFT calculations. The bulk Brillouin zone of cubic symmetry is shown in Fig. 1b with the (110) plane highlighted. Based on this geometry, the energy-dependent electronic band dispersion along 
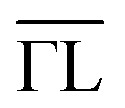
 was analyzed (Fig. 1c) with a binding energy window of more than 1.5 eV. The *E*_F_ shifted from the valence band maximum toward higher energies, displaying a dispersive band minimum at the L point 0.4 eV above the valence band, which could be associated with the surface states; this was confirmed by HAXPES. In addition, no in-gap states were observed, at least for an energy range ∼0.5 eV above the top of the valence bands, suggesting that there was no contribution from in-gap states in p-type single crystals. Furthermore, since no bulk conduction bands were observed, we concluded that the single crystals were p-type—which is consistent with the transport properties of such crystals and will be discussed later—and will focus on the analysis of valence bands below the *E*_F_. The valence bands at L and Γ showed a near-parabolic dispersion around the symmetry points, which mostly originated from the d orbitals of Co atoms, as indicated by the DFT-calculated band structure (Fig. S2†).^26^

Most importantly, both the valence bands at L and Γ show their maxima in a close energy range, 0.5 eV below the *E*_F_ and with a continuum of electronic states of inverted U shape. The negligible energy difference (<0.05 eV) between the L and Γ maxima was smaller than those in the theoretically calculated band structure (Fig. S2,† ∼0.08 eV) and in previous works,^26^ which showed that the valence band maximum at the Γ point had clearly higher energy than the maximum at the L point. Thus, the bands at both points would contribute to the electronic transport in p-type samples, as the thermal energy range at the typical highest-zT temperature of half-Heusler alloys is >0.06 eV (>700 K); therefore, it agreed with the band convergence situation for the p-type material.

Moreover, as shown in Fig. 1d, a double degeneracy at the Γ point was observed, which could further increase the band degeneracy and therefore contribute to a better thermoelectric performance. The effective masses of each pocket were also resolved based on the ARPES results. At the Γ point, there was a heavier valence band with an effective mass of 1.9*m*_e_ and a lighter one of 0.59*m*_e_. Both values were smaller than those predicted for the XCoSb (X = Ti, Zr, Hf) system.^34,35^ However, at point L, the band was more localized with an effective mass of 3.8*m*_e_. The combined total effective mass of both the L and Γ bands would therefore yield a value of around a few *m*_e_, which agrees with the high effective mass in previous reports^7,8,15,26^ and with that obtained from the transport properties of the batch 1 samples below. The simultaneous contribution from low mass bands at a slightly higher energy (Γ point) than the heavier L band is consistent with the description of band convergence enhancing the thermoelectric performance in several systems.^2,36–40^

### The absence of in-gap states in TiCoSb determined by HAXPES

HAXPES was used to determine whether there were in-gap states contributed by Ti or Co interstitials, as it has a significantly larger penetration depth than that of ARPES. TiCoSb was one of the compounds investigated by HAXPES quite early, when HAXPES had a rather low resolution.^21,41^ Studies^41^ resolved the bulk electronic structure and described the in-gap states as a consequence of interstitial defects in polycrystalline, Sc-, and V-substituted TiCoSb samples.^26^

**Fig. 2:**
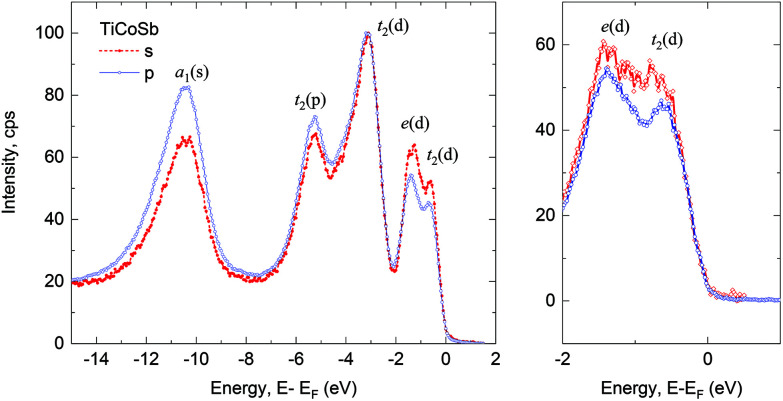
HAXPES valence band spectra of TiCoSb along (001). (a) Spectra taken with s and p polarization of the photons of 6 keV energy (intensity maxima are assigned to the irreducible representations at Γ and the dominating angular momentum character) and (b) high-resolution spectrum close to the Fermi energy. The resolution was set to 150 meV in (a) and 100 meV in (b) by changing the pass energy of the analyzer.

Herein, using TiCoSb single crystals with precise stoichiometry, we aimed to determine their intrinsic point defects. The polarization-dependent valence-band spectra of TiCoSb are shown in Fig. 2. The normal emission direction was nearly parallel to the (001) surface of the single crystal. Photon incidence grazed at 90° with respect to the normal emission direction, and the cleanliness of the sample was analyzed as shown in Fig. S3.†

The photon energy dependence of the cross-sections changes the weights of the emission intensities from the s, p, and d states. At higher energies, the s states had higher intensities, whereas the intensities of the d states were lowered. The most striking result was that no in-gap states were detected in the spectra, demonstrating the negligible Ti or Co interstitials in the single crystals.

### Transport properties of TiCoSb single crystals

**Fig. 3:**
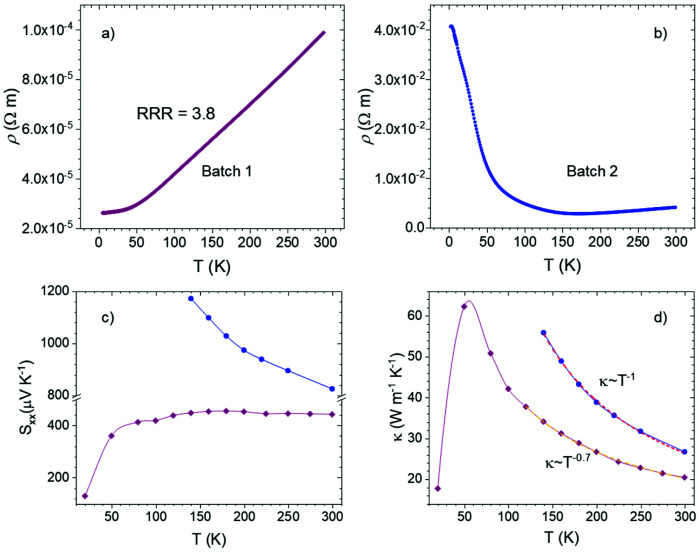
(a) and (b) Electrical resistivities, (c) Seebeck coefficients and (d) total thermal conductivity of TiCoSb single crystals of batch 1 (red diamonds) and batch 2 (blue circles) measured under a four-probe set-up.

Although no in-gap states were observed in the TiCoSb single crystals, a small amount of intrinsic point defects still significantly altered the transport properties. The evolution of the electrical resistivity of the TiCoSb single crystals of two different batches with temperature is shown in Fig. 3a and b. The first batch showed metallic behavior as a steady increase of the resistivity. A residual resistance ratio (RRR) of 3.8 was comparatively large for the half-Heusler system, which suggested that the number of point defects in the sample was comparatively low.^42–44^ In general, the low RRR value of other half-Heusler alloys was attributed to the abundance of crystallographic defects in them, which is common among all alloys belonging to this family.^45^ In contrast, the second batch displayed semiconducting behavior, showing an exponential decrease in resistivity at temperatures between 2 and 150 K, and the resistivity values were two to three orders of magnitude higher than that of the batch 1 sample. This semiconducting behavior was expected for intrinsic TiCoSb systems because of their VEC of 18; this behavior was also determined by theoretical calculations for the defect-free material, which placed *E*_F_ at the valence band maximum.^26^

The Seebeck coefficients and total thermal conductivities of the different batch crystals are shown in Fig. 3c and d, respectively. A positive Seebeck coefficient indicated hole-dominated transport and large absolute values corresponding to low carrier concentrations in the samples. This result was contrary to that reported in the literature of n-type Seebeck coefficients in undoped TiCoSb samples, indicating that the preparation and grain boundaries of polycrystalline samples resulted in n-type defects. Batch 1 crystals displayed increasing Seebeck coefficients, with a nearly independent evolution in the range of 100–300 K, similar to that displayed by doped p-type TiCo_1−*x*_Fe_*x*_Sb.^22^ On the other hand, the Seebeck coefficient of the batch 2 sample decreased with increasing temperature, suggesting a strong contribution from bipolar transport and a deviation from the single carrier behavior at rather low temperatures.

In both batches, the total thermal conductivity represented the lattice contributions, with values as large as the ones expected for undoped pure half-Heusler alloys. They decreased monotonically with a good fit to *T*^−0.7^ and *T*^−1^, in good agreement with acoustic phonon scattering in single-phase materials.^46^ The resistivity evolution and lower thermal conductivity of batch 1 crystals suggested the presence of more defective samples in them than in the batch 2 crystals, which was also supported by the bipolar and semiconducting behavior of the latter.

**Fig. 4:**
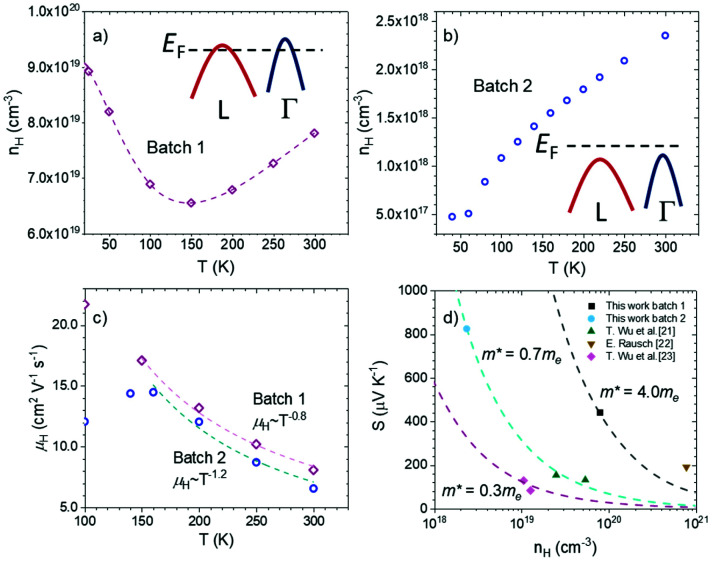
(a) and (b) Hall carrier concentration and (c) Hall carrier mobility of TiCoSb single crystals from two different batches. The dashed line in (a) is a guide for the eyes. (d) Pisarenko plot of p-type TiCoSb derivatives.^22–24^

The collected Hall data displayed the p-type nature of the charge carriers for both batches 1 and 2. The former (Fig. 4a) displayed a hole concentration of ∼7.5 × 10^19^ cm^−3^ at 300 K and almost no variation from 2 to 300 K, similar to that displayed by a degenerate semiconductor. The hole concentration of batch 2, ∼1 × 10^18^ cm^−3^, was much lower (Fig. 4b), but still slightly larger than that of an intrinsic semiconductor. The contradiction between the highly stoichiometric and undefective features shown by the characterization techniques could be due to the relatively low number of defects in the samples and the fact that they may not have altered the overall stoichiometry of the samples. Inspired by the ARPES results and transport properties, we propose that the Fermi level of TiCoSb is easily altered by intrinsic point defects and may vary from an in-gap *E*_F_ to a valence band intersecting *E*_F_, as shown in the insets of Fig. 4a and b for batches 1 and 2, respectively. Although both had stoichiometric compositions, the batch 1 sample had a deeper *E*_F_ and therefore showed a metallic transport behavior and a higher effective mass, contributed by both Γ and L bands. The *E*_F_ of batch 2 lies in the gap, resulting in a semiconducting transport behavior and lighter effective mass, which was only contributed by the Γ band.

Previously reported polycrystalline TiCoSb samples showed slight *n*-character with almost constant temperature-dependent resistivities for the undoped material.^26,34,47^ It has been demonstrated that processing and compacting steps such as ball milling and spark plasma sintering may introduce several defects, either in the crystalline structure or in grain boundaries.^48,49^ These defects alter the electronic transport properties, while the grain boundaries of polycrystalline samples have been shown to limit the scattering length of charge carriers.^9,50–52^ These results agree with a recent theoretical study, which attributed the n-type character of the polycrystalline samples to an increased *E*_F_ and to the Co-interstitial in-gap states, while indicating the p-type behavior of the defect-free materials.^53^

The carrier mobility evolution with temperature (Fig. 4c) displayed acoustic phonon scattering at temperatures above 100 K for batches 1 and 2. It is noteworthy that the mobility values were analogous for both samples, whereas those of batch 1 were slightly larger, despite the higher carrier concentration. This has been observed in other half-Heusler alloys, as the scattering mechanisms may differ at different ranges of the carrier concentration.^54^

The Pisarenko plot shown in Fig. 4d displays the values of previously reported p-type doped TiCoSb, for which there is no consistency in effective mass contributing to the Seebeck coefficient.^22–24^ Even for single crystals, samples from different batches showed very different effective mass values based on the Seebeck coefficients; m* ≈ 4.0*m*_e_ and a rather light mass of 0.7*m*_e_ were determined for batch 1 metallic and batch 2 semiconductive samples in this study, respectively. As such, point defects and in-gap states make it challenging to determine the band features from thermoelectric transport properties as well as the intrinsic electronic band structure through theoretical methods, which have hindered a straightforward understanding of improving the thermoelectric performance of TiCoSb systems. The different effective mass is reflected in the power factor. High resistivity values result in 0.15 mW cm^−1^ K^−2^ for batch 2, whereas batch 1 shows 2.0 mW cm^−1^ K^−2^ at room temperature. Despite the undoped composition, the latter is comparable to that of optimized half-Heuslers with high thermoelectric performance at high temperatures.^55,56^ Thus, it is another indication of the band convergence effect on the high-performance p-type TiCoSb derivatives.

Since small energy differences with the actual electronic structure could have a significant impact on thermoelectric transport, the observations in our study underlined the difficulties in theoretical calculations, especially in cases wherein more than one band contributes to electronic transport. Notably, recalling that different samples showed very different effective masses, ranging from ∼0.3 to ∼4*m*_e_, as determined by their Seebeck coefficients (Fig. 4d), we propose that different Fermi surfaces dominate the transport behavior from sample to sample. In general, the transport behavior in samples with low effective masses is mainly attributed to the Fermi surfaces at the Γ point, while the L band (or both L and Γ bands) dominates in the samples with high effective masses. This is possible because the energy difference between the L and Γ maxima is negligible (<0.05 eV). We further argue that the different Fermi surface-dominated transport features are due to the various point defects in the XCoSb system.

## Conclusions

The intrinsic electronic structures of half-Heusler TiCoSb samples were determined using ARPES and HAXPES in single crystals. XRD displayed only the half-Heusler *F*4̄3*m* structure and the compositional analyses suggested an almost perfect stoichiometry with no signs of any excess of Ti or Co. A band convergence scenario was determined by ARPES for the L and Γ band maximum points, with three different bands contributing within a small energy range, providing further insight into the excellent electronic performance of this family of materials. Both the ARPES and HAXPES spectra discarded the presence of occupied in-gap states above the valence-band maximum. Although, in the absence of in-gap states, the concentration of any defects should be rather low, the Fermi level and the transport properties were found to be very sensitive to intrinsic point defects. Electrical resistivity measurements of two separately synthesized crystals showed semiconducting and metallic behaviors, respectively, with different charge carrier concentrations from 10^18^ to 10^19^ cm^−3^. Therefore, we concluded that subtle changes in the crystallographic structure owing to intrinsic point defects could lead to dramatic alterations at the Fermi level, creating changes in the electrical properties of the crystals. Future work aimed at enhanced thermoelectric performance of half-Heusler TiCoSb will be better suited to use its band convergence feature and also focus on the point defect chemistry in this system.

## Author contributions

F. S., M. Y., E. L., C. F., and Y. P. participated in conceptualization, F. S., M. Y., G. F., A. G., G. A., U. B., B. H., D. C., A. F., E. L., and C. F. participated in the investigation, and the manuscript was written through contributions of all authors and all authors have given approval to the final version of the manuscript.

## Conflicts of interest

There are no conflicts to declare.

## Supplementary Material

NR-014-D2NR02556F-s001
