# Supplementary material for: Electronic structure and low-temperature thermoelectric transport of TiCoSb single crystals
Source: Nanoscale. 2022 Jun 21;14(28):10067–74. doi: 10.1039/d2nr02556f (PMC9302267; doi:10.1039/d2nr02556f)
Supplement: NR-014-D2NR02556F-s001 [file NR-014-D2NR02556F-s001.pdf]

Electronic Supplementary Material (ESI) for Nanoscale.
This journal is © The Royal Society of Chemistry 2022

# Electronic structure and low-temperature thermoelectric transport of TiCoSb single crystals

Federico Serrano-Sanchez[a†], Mengyu Yao[a], Bin He[a], Dong Chen[a], Andrei Gloskovskii[b], Alexander Fedorov[c,d], Gudrun Auffermann[a], Enke Liu[e], Ulrich Burkhardt[a], Gerhard H. Fecher[a], , Chenguang Fu[a,f], Claudia Felser[a], Yu Pan[a†]

[a.] *Max Planck Institute for Chemical Physics of Solids, 01187 Dresden, Germany*
[b.] *Deutsches Elektronen-Synchrotron DESY, 22607 Hamburg, Germany*
[c.] *Helmholtz-Zentrum Berlin für Materialien und Energie, Berlin, Germany*
[d.] *Institute for Solid State Research, Leibniz IFW Dresden, 01069 Dresden, Germany*
[e.] *Beijing National Laboratory for Condensed Matter Physics, Institute of Physics, Chinese Academy of Sciences Beijing, 100190 (P. R. China)*
[f.] *State Key Laboratory of Silicon Materials, School of Materials Science and Engineering, Zhejiang University, 310027 Hangzhou, ChinaAddress here.*
† Serrano@cpfs.mpg.de; Yu.Pan@cpfs.mpg.de

Table S1. Structural parameters of TiCoSb batch 1 crystals from XRD refinement.

| **Cubic, Space group $F\bar{4}3m$** | | | |
|---|---|---|---|
| *Hall symbol* | F -4 2 3 | *X-Ray Wavelength* | 1.54060 Å |
| *a(Å)* | 5.8774(1) | *Sample morphology* | powder |
| *V(Å3)* | 203.030(4) | *Preparation* | Ground crystals |
| *Z* | 4 | | |
| **Rietveld refinement** | | 91 parameters | |
| *Profile Function* | Pseudo-Voigt | $R_I$ | 2.39 |
| *Background profile* | Linear interpolation between a set of background points with refinable heights. | $R_{wp}$ | 9.12 |
| *Excluded regions* | No | $R_{exp}$ | 1.76 |

| Atomic parameters | x | y | z | $U_{iso}$ | Occ. (<1) |
|---|---|---|---|---|---|
| **Ti** | 0.00000 | 0.00000 | 0.00000 | 0.0090(6) | 0.933(3) |
| **Co** | 0.25000 | 0.25000 | 0.25000 | 0.0117(6) | 0.950(3) |
| **Sb** | 0.50000 | 0.50000 | 0.50000 | 0.0056(3) | 1.0 |

Fig. S1. Back scattered electron SEM images of TiCoSb crystal pieces for wavelength-dispersive X-ray spectroscopy (WDX). The pieces are polished down to thin layers prior to elemental analysis.

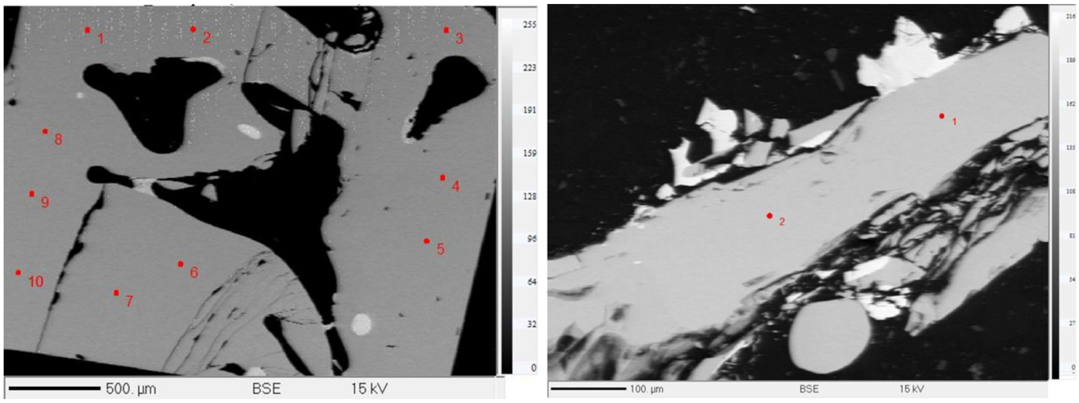


Table S2. Elemental analysis carried out by WDX averaged for ten different points in the crystals. Total un-normalized weight is an indication of the results reliability.

| **Element** | **Un-normalized Weight %** | **At. %** | **Un-normalized Weight %** | **At. %** |
|---|---|---|---|---|
| | Batch 1 | | Batch 2 | |
| **Ti** | 21.4(1) | 33.8(1) | 21.4(1) | 33.6(1) |
| **Co** | 25.9(1) | 33.2(1) | 26.3(1) | 33.6(1) |
| **Sb** | 53.1(1) | 32.9(1) | 53.0(1) | 32.8 (1) |
| **Total** | 100.54 | | 100.76 | |

Figure S2. Electronic structure of TiCoSb and theoretical spectra of HAXPES with polarized light.

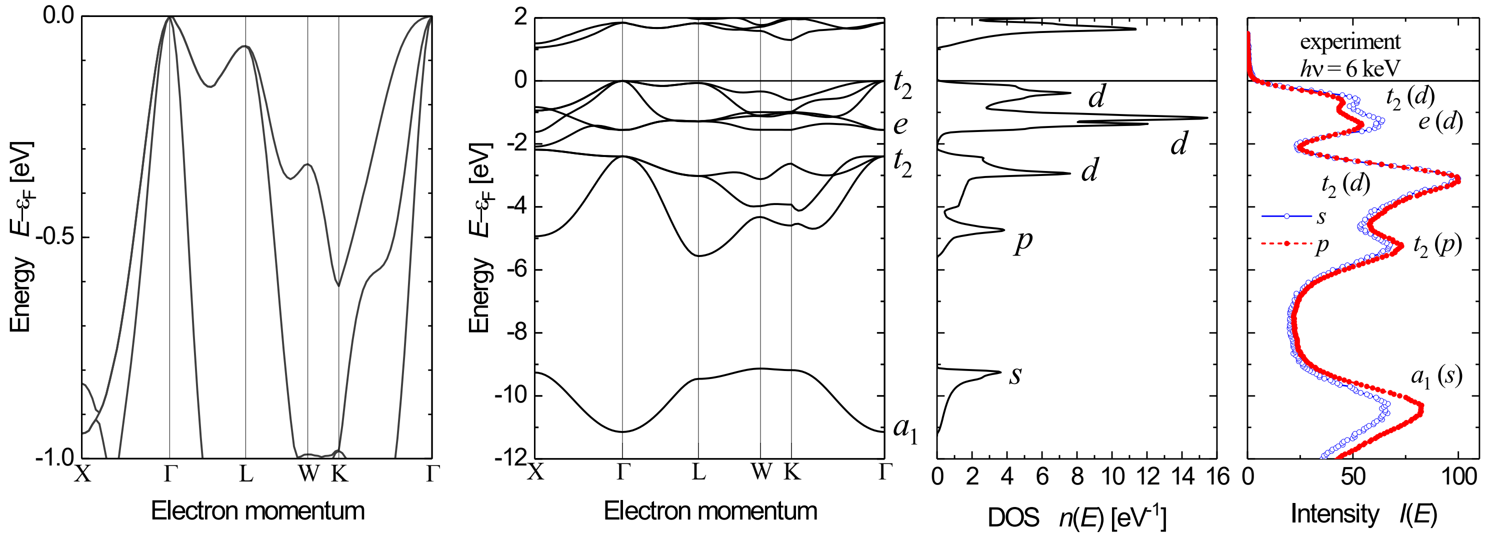


Figure S3. Sb 3d Core level spectra of CoTiSb. Plasmon losses are assigned with ωp.

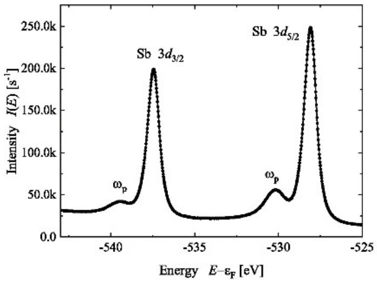


*Sample cleanliness.*

The cleanliness of the sample used in the HAXPES measurements was monitored by taking survey spectra over an energy range of 1 keV and, in particular, spectra in the energy range of the Sb 3*d* and O 1*s* core states as shown in Figure S3. No signature of the oxygen 1*s* core level was detected, demonstrating that the sample is clean and not oxidised. The Sb 3*d* core level exhibit, besides a plasmon splitting (~ωp = 2.1 eV), a spin orbit splitting of ΔSO =8.9 eV.
